# Confirming the identity of two enigmatic “spiny solanums” (Solanum subgenus Leptostemonum, Solanaceae) collected by Jean-Baptiste Leschenault in Java

**DOI:** 10.3897/phytokeys.70.9758

**Published:** 2016-10-04

**Authors:** Xavier Aubriot, Caroline Loup, Sandra Knapp

**Affiliations:** 1 Department of Life Sciences, Natural History Museum, Cromwell Road, London SW7 5BD, UK; 2 Herbier de l’Université de Montpellier, Service du Patrimoine Historique - DCSPH - CC99010, 163 rue Auguste Broussonnet, 34090 Montpellier, France

**Keywords:** Exploration, Jean-Baptiste Leschenault de la Tour, Indonesia, Montpellier, Nicolas Baudin, Toussaint François Node-Véran, typification

## Abstract

Taxonomic revision of the tropical Asian species of *Solanum* revealed two names, *Solanum poka* Dunal and *Solanum graciliflorum* Dunal, whose identities were uncertain and whose application has always been tentative. Material collected in Java at the beginning of the 19^th^ century by Jean-Baptiste Leschenault de la Tour and used to describe these taxa has not been found, despite extensive searches in European herbaria. We here stabilise use of these names by comparing herbarium specimens and drawings of original material made by the artist Toussaint François Node-Véran. Detailed descriptions with synonymy, preliminary conservation assessments and specimen citations are provided for both species. Lectotypes are designated for all names (including synonyms) and epitypes designated for *Solanum poka* and *Solanum graciliflorum* to stabilise usage.

## Introduction

In 1800, shortly after he became First Consul of the Republic of France, Napoléon Bonaparte approved an expedition along the “coasts of New Holland” (Australia). The expedition, led by Nicolas Baudin, has been cited as one of the most ambitious and the most enriching for collections of natural history of the great scientific expeditions of the early 19^th^ century (Cornell 1965, Fornasiero et al. 2010). Naturalists brought back from these distant and previously unexplored lands many new plant species, both as herbarium specimens and as living plants or seeds that were grown out mostly in the plant beds and greenhouses of the Muséum National d’Histoire Naturelle of Paris and in Josephine Bonaparte’s gardens at Malmaison (Jangoux 2004, Fornasiero et al. 2010).

The Baudin expedition lasted four years (1800-1804) and its explicit purpose was “observation and research relating to Geography and Natural History”. The crew included 24 scientists and artists, among them were three botanists and five gardeners that had been carefully selected by Antoine-Laurent de Jussieu, then director of the Muséum National d’Histoire Naturelle (Proust de la Gironière 2002, Jangoux 2004). By the time the *Géographe* and the *Naturaliste* reached Port Jackson (New South Wales) in June 1802 for a five month stopover, most of the botanical team had either died or left the expedition; only one botanist, Jean-Baptiste Leschenault de la Tour, and one gardener, Antoine Guichenot, remained (Desmet and Jangoux 2010). After collecting in Australia and continuing with the expedition, in 1803 Leschenault was left behind in Timor to recover from illness (Proust de la Gironière 2002, Desmet and Jangoux 2010). After his recovery, he left Timor for Java, but found himself unable to return to France, probably due to instability in Europe at the time. Leschenault was offered the protection of Nicolous Engelhard, the Dutch Governor of the northeastern coast of Java, and given the mandate to collect natural history specimens there (Van Steenis-Kruseman and Van Steenis 1950, Desmet and Jangoux 2010). For two years (1804-1806) Leschenault visited the islands of Java and Madoera where he claimed to have collected ca. 900 plant species (Leschenault 1807), all of which were presumably sent back to the herbarium of the Muséum National d’Histoire Naturelle in Paris (P). Several duplicates of Leschenault’s collections in other groups have been found in G, K and L (Van Steenis-Kruseman and Van Steenis 1950), but no catalogue of his collections exists and an accurate estimate of the extant number of collections has yet to be compiled.

In the course of preparing a monographic revision of the spiny solanums from tropical Asia (see Aubriot et al. 2016 for discussion of the Old World clade of subgenus Leptostemonum Bitter), we were unable to find the type material for two spiny solanums from Java. *Solanum graciliflorum* Dunal and *Solanum poka* Dunal were first described by Michel-Félix Dunal in 1814 as part of the supplement of Lamarck’s *Encyclopédie Méthodique* edited by Jean Poiret. He cited no herbarium material or collector but cited a drawing (“*Dun. Suppl. Sol. tab.*”; Dunal 1814) from his then unpublished synopsis of *Solanum* (published later as Dunal 1816). In later treatments of these species Dunal (1816, 1852) stated that the collections he had seen were made by Leschenault during his stay in Java (1803-1806). Thorough searches of the herbarium at P where Leschenault’s collections are housed, as well as other herbaria (see Materials and Methods) where duplicates could possibly have been sent, have not revealed any original material upon which the drawings cited in the protologue were based. Toussaint François Node-Véran was the official botanical artist of the Jardin des Plantes in Montpellier in the early part of the 19^th^ century (appointed in 1813 and stayed there until his death in 1852; Denizot et al. 1994) and worked closely with Dunal in preparing the illustrations for the intended major treatment of the taxonomy of *Solanum* (Knapp 2007). Several hundred pen and ink drawings of *Solanum* were made by Node-Véran during the preparation of Dunal’s complete treatment of the genus that was never published in its entirety, but only as *Solanorum Synopsis* (Dunal 1816). Political instability in France during the years of the Napoleonic Wars of the early 19^th^ century and Dunal’s not being appointed director of the Jardin des Plantes in Montpellier could be contributing factors in his failure to publish the complete illustrated volume (Dulieu 1994, Knapp 2007). Several of the species drawn by Node-Véran were drawn directly from herbarium specimens [e.g., *Solanum arboreum* Dunal, *Lycopersicon hirsutum* Dunal (=*Solanum habrochaites* S.Knapp & D.M.Spooner); see Knapp and Spooner 1999, Knapp 2007] that are currently in the herbarium at P. We expect he similarly used herbarium material from P (explicitly cited as herbarium material in Dunal 1816) as the basis for the illustrations of *Solanum graciliflorum* and *Solanum poka* cited in the 1814 protologues (Dunal 1814). It is possible that specimens were lost during the turbulent times in Europe in the early 19^th^ century (see Knapp 2007).

Given that no plant specimens corresponding to the protologues have been found, despite extensive searches, we consider the unpublished Node-Véran drawings the most appropriate and only extant possibilities for lectotypifying both *Solanum graciliflorum* and *Solanum poka*. These two names have long been treated as confusing, or ignored; they have rarely been used (see below in each species treatment), and few herbarium specimens we have seen have been annotated with either name. Most specimens of the taxa we here recognise as *Solanum graciliflorum* and *Solanum poka* have been annotated incorrectly as widespread weedy taxa (e.g., *Solanum torvum* Sw.) or with names we here consider synonyms (e.g., *Solanum athroanthum* Dunal); this reflects the limited taxonomic work previously done on tropical Asian *Solanum*, whose taxonomy has not been revised in detail since Dunal’s (1852) treatment for Candolle’s *Prodromus*. Our purpose here is to secure the application of these names by designating lectotypes for *Solanum graciliflorum* and *Solanum poka*, as well as providing complete morphological descriptions for these two species. We also designated interpretative types (epitypes), because details of trichome morphology are extremely important in spiny solanum taxonomy, and these are not visible on the illustrations.

## Materials and methods

Searches for type specimens of *Solanum graciliflorum* and *Solanum poka* were made using the resources available in Global Plants (http://plants.jstor.org/) and physically in the herbaria where duplicates could possibly be kept (A, BM, E, G, K, L, LE, MPU and P; abbreviations follow Index Herbariorum; http://sweetgum.nybg.org/science/ih/). Complete details for all specimens examined here are in the data supplement to this article (Suppl. material 1). Morphological descriptions are based on herbarium specimens; we have seen all specimens cited here. Geographical coordinates have been calculated using Google Earth (https://www.google.com/earth/) if not already recorded on specimens.

## Taxonomic treatment

### Solanum graciliflorum

Taxon classificationPlantaeSolanalesSolanaceae

Dunal, Encycl. [J. Lamarck & al.] Suppl. 3: 763. 1814.

Fig. 1a, b

Solanum athroanthum Dunal, Prodr. [A. P. de Candolle] 13(1): 208. 1852. Type. Indonesia. Java: [Prov. Banjinwanyne] “in sylvis prope Sukaradja” [Sukaraja], 1846, *H. Zollinger 2907* (lectotype, designated here: G-DC [G003043306]; isolectotypes: G-DC [G00301684], BM [BM000778325], MPU [MPU012648], P [P00368939, P00368940, P00368941]).

#### Type.

Based on an unpublished illustration of Leschenault collection kept in the Node-Véran collection in Montpellier (lectotype, designated here: Service du Patrimoine Historique de l’Université de Montpellier Node-Véran, Sol. Tab. 47 [MPU028534]); Indonesia. East Java: Blambangan [Sumberwaru, Badjulmati], *T. Horsfield s.n.* (epitype, designated here: BM [BM000886121]).

#### Description.

Scandent shrub to 2 m, armed. Young stems terete, brownish grey, very sparsely stellate-pubescent and prickly, the stellate trichomes porrect, sessile to subsessile, the rays (4-)5–8, 0.1–0.25 mm long, the midpoints to 0.15 mm long, the prickles to 7 mm long, to 8 mm wide at base, curved, deltate, laterally flattened, pale yellow, glabrous; bark of older stems dark brownish grey, glabrescent. Sympodial units difoliate, the leaves geminate, usually similar in size. Leaves simple, the blades (4.5-)7–11 cm long, (1.5-)3–5 cm wide, ca. 2 times longer than wide, elliptic to ovate, chartaceous, slightly discolourous; adaxial and abaxial surfaces sparsely to very sparsely stellate-pubescent and usually with at least some prickles, the stellate trichomes porrect, sessile to subsessile, the rays 6–8, 0.1–0.25 mm long, the midpoint to 0.25 mm long, usually as long as the rays, the prickles 0–10(-12) per leaf side, mostly inserted on the midvein, to 9 mm long, to 2 mm wide at base, straight or slightly curved at the tip, awl-shaped, conical, pale yellow, glabrous; major veins 3–4 pairs drying dark; base attenuate to truncate; margins shallowly to deeply lobed, the lobes 1–3 on each side, 0.5–2.5 cm long, broadly deltate, apically rounded, the sinuses extending up to 2/3 of the distance to the midvein; apex rounded to acute; petiole 0.5–1.8 cm long, 1/10–1/6 of the leaf blade length, sparsely stellate-pubescent with porrect, subsessile trichomes denser at the very base, with 0–2 prickles like those of the blades. Inflorescences leaf-opposed or apparently lateral and borne between leaf pairs, 2–4 cm long, unbranched to up to 6 times branched, with 15–50+ flowers; axes sparsely to very sparsely stellate-pubescent, unarmed; peduncle 1–2(-2.5) cm long, with 0–1 prickles like those of the leaves and stems; pedicels 4–7 mm long, erect, articulated at the base, very sparsely stellate-pubescent, unarmed; pedicel scars spaced 1–5 mm apart. Flowers 5-merous, apparently all perfect. Calyx 1.75–2 mm long, campanulate, pubescent with sessile porrect stellate trichomes like those of the stems, unarmed, the lobes 0.25–0.5 mm long, deltate, apically acute. Corolla 0.5–1 cm in diameter, white to pale lilac, stellate, lobed nearly to the base, the lobes 4–5 mm long, ca. 1 mm wide, narrowly deltate to linear, reflexed at anthesis, densely stellate-pubescent abaxially, the trichomes porrect, sessile, the rays 4–6, 0.1–0.2 mm long, the midpoints the same size than the rays or to 0.25 mm long. Stamens slightly unequal; filament tube < 0.5 mm long; free portion of the filaments almost equal, 0.5–1.25 mm long; anthers unequal, three of the five 4.5–5 mm long and two 3–4 mm long, all 0.5–0.75 mm wide, glabrous, connivent, tapering, poricidal at the tips, the pores not lengthening to slits with age. Ovary conical, minutely glandular-puberulent; style ca. 5.5 mm long, slender, curved at the apex, glabrous; stigma capitate, minutely papillate. Fruit a globose berry, 6–50+ per infrutescence, 3–5 mm in diameter, the pericarp shiny, red when mature, glabrous; fruiting pedicels 0.8–1.2 cm long, ca. 0.5 mm in diameter at the base, tapering to a slightly enlarged apex, woody, spreading, unarmed; fruiting calyx lobes slightly expanding to 1.5 mm long, ca. 1/5 the length of the mature fruit, deltate to lanceolate, unarmed. Seeds 6–9 per berry, 3.5–4 mm long, 3–3.5 mm wide, flattened-reniform, orange-brown, the surface minutely pitted, the testal cells pentagonal in outline.

**Figure 1.**
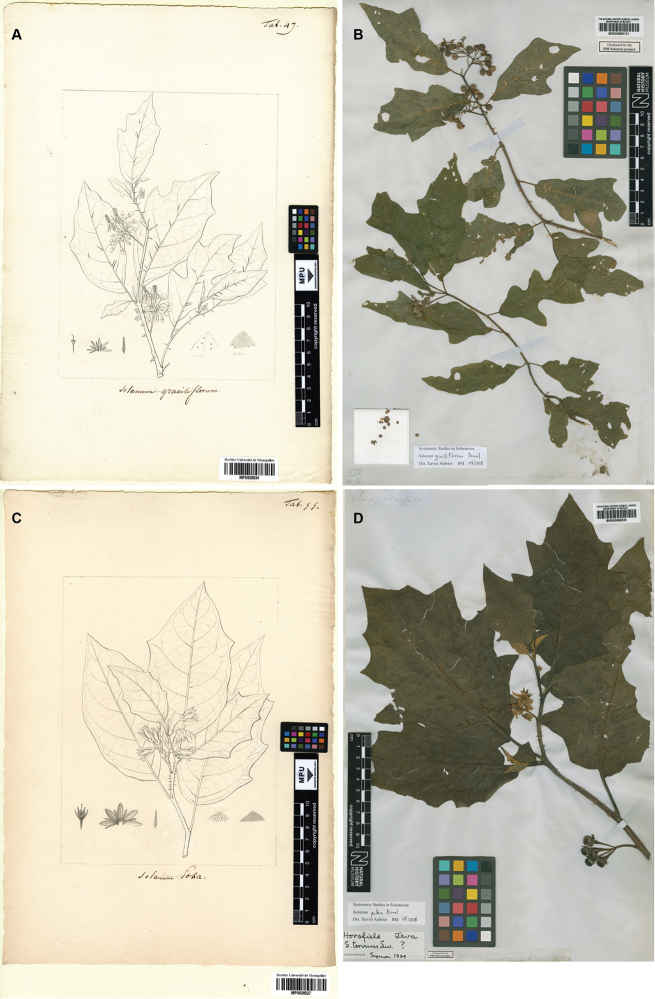
**A** Lectotype of *Solanum graciliflorum*, illustration *Sol. Tab. 47* [MPU028534] made by T.F. Node-Véran (1773–1852). Reproduced with permission of the Université de Montpellier – Herbier MPU (Service de Patrimoine Historique); copyright Université de Montpellier – Herbier MPU (SPH) **B** Epitype of *Solanum graciliflorum*, *T. Horsfield s.n.* [BM000886121] **C** Lectotype of *Solanum poka*, illustration *Sol. Tab. 55* [MPU028527] made by T.F. Node-Véran (1773-1852). Reproduced with permission of the Université de Montpellier – Herbier MPU (Service de Patrimoine Historique); copyright Université de Montpellier – Herbier MPU (SPH) **D** Epitype of *Solanum poka*, *T. Horsfield s.n.* [BM000886306].

#### Phenology.

The few known collections were flowering and fruiting between May and August.

#### Distribution and ecology.

**Figure 2.**
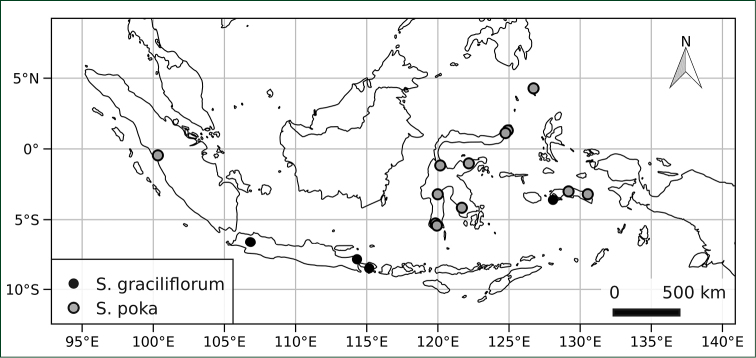
Distribution of *Solanum graciliflorum* and *Solanum poka* in the Malay Archipelago. Geographical information for these collections can be found in the data supplement to this article (Suppl. material 1).

(Fig. 2) Known from the islands of Java, Bali, Sulawesi and Ambon (Indonesia); growing in forest understory; elevation not recorded on any herbarium material we have seen. The records (as *Solanum athroanthum*) from the island of Luzon in the Philippines (Merrill 1912, Merrill 1923) are based on misidentifications of specimens of *Solanum trilobatum* L.

#### Preliminary conservation status.


 Data Deficient (DD); known only from seven collections, several of which are of uncertain localities. *Solanum graciliflorum* has not been re-collected since the first half of the 20^th^ century, indicating it is certainly of conservation concern. Recollection of this species and exploration of the type locality are priorities.

#### Specimens examined.

**Indonesia. Bali**: Perepat Agoeng, 21 Jul 1934, *de Voogd 2177* (A); **Gorontalo**: North Celebes, Jun 1875, *Riedel s.n.* (K); **Java**: sin loc., 1802, *Horsfield 15* (K); West Java, Bogor, *Anonymous s.n.* (K); **Malaku**: “Malay Archipelago, Dawalore [Ambon, Dawa-lour]”, Aug 1883, *Riedel s.n.* (K).

#### Discussion.

*Solanum graciliflorum* is a poorly known species represented by very few collections that presents a combination of morphological features that makes it readily recognisable among tropical Asian spiny solanums. It is superficially similar to *Solanum cyanocarphium* Blume, a sympatric species that is distributed across the Sunda Shelf region, and to *Solanum retrorsum* Elmer, that occurs mainly in the Philippines. *Solanum graciliflorum* can be distinguished from both of them by its much sparser indumentum, stout, deltate stem prickles (rather than slender and awl-shaped), and tiny, delicate flowers (hence the species epithet) that are clustered in dense, many-flowered inflorescences. Molecular data show that *Solanum cyanocarphium* and *Solanum graciliflorum* are not closely related; *Solanum graciliflorum* is nested within the Sahul-Pacific clade while *Solanum cyanocarphium* is an unresolved species of uncertain affinities (see Aubriot et al. 2016).

*Solanum graciliflorum* is the type of section Graciliflorum (Dunal) Seithe, a section partly based on the informal grouping made in Dunal’s (1852) treatment of *Solanum* in Candolle’s *Prodromus*. In Seithe’s (1962) circumscription, section Graciliflorum included 14 species with stellate trichomes and acicular prickles coming from various region of the world (e.g., *Solanum bahamense* L. from the Caribbean archipelago, *Solanum nienkui* Merr. & Chun from Southeast Asia, *Solanum paniculatum* L. from South America, *Solanum stelligerum* Sm. from Australia). Symon (1981, 1985) extended the circumscription of the section with the addition of 27 additional species (10 from Australia and 17 from New Guinea), expressing at the same time serious doubts about its coherence. Symon’s concerns echoed those expressed in Whalen’s systematic treatment of the spiny solanums (Whalen 1984). In this first-ever attempt to include spiny solanums into a morphologically based phylogenetic framework, Whalen did not regard section Graciliflorum as a natural group and placed members of the section as defined by Seithe (1962) into several of his informal groups (e.g., *Solanum bahamense* in the ‘*Solanum bahamense* group’, *Solanum paniculatum* in the ‘*Solanum torvum* group’, *Solanum stelligerum* in the ‘*Solanum ferocissimum* group’). With limited sampling and knowledge of Old World taxa, Whalen did not clarify the identity of *Solanum graciliflorum*, the type species of the section, and included it in his ‘Unusual species group’ as a possible synonym of the widespread tropical Asian species *Solanum violaceum* Ortega. He considered *Solanum athroanthum* to be different from *Solanum graciliflorum*, and placed the former into his ‘*Solanum dunalianum* group’ [= Solanum section Dunaliana (Bitter) Symon *pro parte*], a group of 20 species distributed across the Malayan archipelago, Australia and the South Pacific that were characterised by lack of broad-based prickles on mature growth, entire leaves with glabrate abaxial surfaces, inflorescences with tightly spaced hermaphroditic flowers, and juicy red berries (Whalen 1984). More recently McClelland (2012) proposed a narrower circumscription of sect. Dunaliana, reducing it to six species and excluding *Solanum graciliflorum* (as *Solanum athroanthum*) on the basis of its deeply lobed leaves with prickles on the principal veins and its slightly unequal anthers (versus entire to shallowly lobed non-prickly leaves and always equal anthers for all species he recognized as belonging to sect. Dunaliana). Instead he suggested a close relationship between *Solanum graciliflorum* and *Solanum nienkui*, a Southeast Asian species that also displays anisandry. Recent molecular phylogenetic analysis of tropical Asian spiny solanums incorporating representatives of sections *Dunaliana* and *Graciliflorum* (including *Solanum dunalianum* Gaudich. and *Solanum graciliflorum*) showed *Solanum graciliflorum* to be sister to the Philippine endemic *Solanum lianoides* Elmer (Aubriot et al. 2016). Both species are prickly vines, but *Solanum lianoides* differs from *Solanum graciliflorum* by its denser leaf indumentum, entire leaves and larger flowers. Both species are closely related to *Solanum dunalianum* (Aubriot et al. 2016), a result consistent with Whalen’s (1984) treatment of *Solanum graciliflorum* (as *Solanum athroanthum*; see Aubriot et al. 2016 for discussion) but not with McClelland’s (2012) hypothesis of relationships.

In the protologue Dunal referred to an illustration made by Node-Véran, ‘*Dun. Suppl. 7. Sol. Mss. tab. 4.*’, an orthographic error for ‘*Dun. Suppl. Sol. Mss. tab. 47.*’ according to the sequence of figure numbers and to the caption on the illustration in Montpellier. We were unable to find any herbarium material matching the illustration in either P or MPU, although Dunal later (Dunal 1816, 1852) cited Leschenault as the collector of the material he had seen. We designate the unpublished illustration of Node-Véran as the lectotype because it is the only extant original material we have identified to date. We have also designated here an epitype specimen that best matches Node-Véran’s illustration, and that corresponds to a collection made in the same geographical area as the lost type specimen (i.e. the island of Java in Indonesia) in order to secure the application of the name (Art. 9.8, McNeill et al. 2012).

Dunal (1852) based his description of *Solanum athroanthum* on *Zollinger 2907* in “hb. DC.”. There are two specimens of *Zollinger 2907* in G-DC; we select the more complete of these as the lectotype. The locality data for Zollinger’s collections are often not written on all duplicates; for *Zollinger 2907* locality data are only found on P00368940.

### Solanum poka

Taxon classificationPlantaeSolanalesSolanaceae

Dunal, Encycl. [J. Lamarck & al.] Suppl. 3: 768. 1814.

Fig. 1c, d

Solanum torvum Sw. var. scabrescens Miq. Fl. Ned. Ind. 2: 648. 1861. Type. Indonesia. Sumatra: sin. loc., *F.W. Junghuhn s.n.* (holotype: L [L0403917])

#### Type.

Based on an unpublished illustration of Leschenault collection kept in the Node-Véran collection in Montpellier (lectotype, designated here: Service du Patrimoine Historique de l’Université de Montpellier, Node-Véran, Sol. Tab. 55 [MPU028527]); Indonesia. Java: sin. loc., *T. Horsfield s.n.* (epitype, designated here: BM [BM000886306]).

#### Description.

Shrubs to 3 m, armed. Young stems terete, black to dark brownish, moderately stellate-pubescent, usually densely prickly distally, sometimes unarmed, the stellate trichomes porrect, sessile or variously stalked, the stalks to 0.2 mm long, the rays (4-)5–8, 0.1–0.25 mm long, the midpoints reduced to globular glands; prickles to 3.5 mm long, to 2.5 mm wide at base, straight, awl-shaped to deltate, conical, pale yellow, glabrescent; bark of older stems brownish gray, sparsely stellate-pubescent. Sympodial units difoliate, the leaves geminate. Leaves simple, the blades 11–24 cm long, 4–13 cm wide, ca. 1.5–3 times longer than wide, elliptic to broadly ovate, chartaceous, slightly discolorous; adaxial surface moderately stellate-pubescent with porrect, sessile and less often variously stalked trichomes, the stalks to 0.1 mm long, the rays 4–8, 0.1–0.4 mm long, the midpoints to 0.25 mm long; abaxial surface moderately stellate-pubescent with trichomes like those of the adaxial surface, but more often stalked; prickles 0–6 per leaf side, to 6 mm long, to 1.5 mm wide at base, straight or slightly curved at the tip, awl-shaped, conical, pale yellow, glabrous; major veins 6–8 pairs drying yellow; base shortly attenuate to truncate; margins entire or shallowly to deeply lobed, the lobes 1–5 on each side, 0.5–5 cm long, rounded to apically acute, the sinuses extending up to 2/3 of the distance to the midvein, deltate; apex acute; petiole 1.5–4 cm long, 1/10–1/5 of the leaf blade length, densely stellate-pubescent with porrect, sessile trichomes like those of the blades, with 0–5 prickles like those of the stems. Inflorescences apparently lateral or leaf opposed, 2–5 cm long, unbranched to up to 2 times branched, with ca. 5–20 flowers, moderately to densely stellate-pubescent, unarmed; peduncle 0.5–1.5 cm long, with 0–1 prickles; pedicels 0.5–1.2 cm long, erect, articulated at the base, densely stellate-pubescent, unarmed; pedicel scars spaced 2–4 mm apart. Flowers 5-merous, apparently all perfect. Calyx 4–7 mm long, campanulate, moderately stellate-pubescent, densely stellate-pubescent on the midvein, unarmed, the lobes 3–5 mm long, the lower part deltate and abruptly constricting to an elongate acumen, the acumen 3/4 the total lobe length, the abaxial surface more or less strongly keeled along the midvein. Corolla 1–2 cm in diameter, white, lobed for ca. 1/2–2/3 of the way to the base, the lobes 5–8 mm long, 2–3.5 mm wide, deltate, spreading at anthesis, densely stellate-pubescent abaxially on parts exposed in bud. Stamens equal; filament tube < 0.5 mm long; free portion of the filaments 0.75–1.5 mm long; anthers 5–6.5 mm long, ca. 0.75 mm wide, connivent, tapering, poricidal at the tips, the pores not lengthening to slits with age. Ovary conical, minutely glandular-puberulent; style 0.6–1 cm long, slender, curved at the apex, with few scattered hairs at the tip; stigma capitate, minutely papillate, stellate-pubescent. Fruit a globose berry, 8–18 per infrutescence, 0.8–1.5 cm in diameter, the pericarp smooth, bluish green when young turning to dark greyish yellow, glabrous; fruiting pedicels 1.2–2.5 cm long, ca. 1–1.5 mm in diameter at the base, ca. 2–3 mm in diameter at the apex, woody, erect, unarmed; fruiting calyx lobes not expanding. Seeds 100–200 per berry, ca. 1.75–2 mm long, 1.5–1.75 mm wide, flattened reniform, pale yellowish, the surface minutely pitted, the testal cells sinuate in outline.

#### Phenology.

Flowering and fruiting throughout the year.

#### Distribution and ecology.

(Fig. 2) Widely distributed in the Malay Archipelago, from western Sumatra to the Maluku Islands and across Sulawesi, northwards to the Talaud islands; growing in open woodland, forest edges, degraded vegetation, usually on limestone or volcanic rocks; 0–1600 m elevation.

#### Preliminary conservation status.


 Least Concern (LC); EOO > 100,000 km^2^ and AOO > 10,000 m^2^ (see Moat 2007 for explanation of measurements). Although the EOO and AOO measurement indicate a status of least concern, the few collections coupled with the profound transformation in lowland Indonesian habitats where *Solanum poka* is found (Margono et al. 2014) suggest that the species is a priority for recollection and reassessment.

#### Specimens examined.

**Indonesia. Central Sulawesi**: Banggai regency, Luwuk District, Bunta Subdistrict, Sumber Agung, Gunung Hek, Sungai Hek, Cabang Tiga, 980 m, 27 Feb 2004, *Hendrian et al. 964* (E, L); Sigi Regency, near the river S of Tongoa, 650 m, 17 Mar 1981, *Johansson et al. 419* (K, L); **Java**: sin. loc., *Horsfield s.n.* (BM); sin. loc., *Horsfield 786* (BM); **Malaku**: Central Maluku Regency, Wae Mamahala, 1330 m, 11 Nov 1937, *Eyma 2166* (A, L); Central Maluku Regency, Seram Utara District, Manusela National Park, along a trail from Wae Puo to Kali, Ili area, south of Sawai, 830–1230 m, 23 Jan 1985, *Kato et al. C-5431* (A, L); East Seram Regency, Bula District, Luman, 15 km south of Bula, 10–20 m, 26 Feb 1985, *Kato et al. C-7942* (L); **North Sulawesi**: Minahasa Regency, Mt. Soputan, 1080 m, 11 Oct 1973, *de Vogel 2504* (L); Minahasa Regency, Tondano, 1840, *Forsten s.n.* (L); Minahasa Regency, 25 Apr 1895, *Koorders 18035B* (L); Minahasa Regency, 20 m, 28 Apr 1895, *Koorders 18037B* (L); Talaud Islands Regency, Pulau Karakelang, bank of Kuala Bahewa, 30 m, 3 May 1926, *Lam 2772* (K, L); **South Sulawesi**: Gowa Regency, Lombasang, 1000 m, 26 May 1921, *Bunnemeyer 11732* (K, L); Gowa Regency, Lombasang, 1100 m, 31 May 1921, *Bunnemeyer 11813A* (L); Bantaeng Regency, Bonthain [Bantaeng], 1500 m, 12 Jun 1921, *Bunnemeyer 12117* (L); Kolaka Regency, Baula, 150 m, 26 Dec 1909, *Elbert 3224* (L); Enrekang Regency, Enrekang District, Latimojong Mts., in valley 3 km. south west of Bunte Tjejeng and south east of Rantelemo, 1490 m, 14 Nov 1969, *Sands 477* (A, E, K); **Timor**: sin loc., 1882, *Forbes 3806* (BM, L); **West Sumatra**: Agam Regency, Mt. Singgalang, 1600 m, 29 May 1918, *Bunnemeyer 2786* (A, L).

#### Discussion.

*Solanum poka* was long ignored after its first publication (Dunal 1814). It has not been included in classical floristic treatments of Java (Hasskarl 1848, Backer 1965, van Steenis et al. 2006) or Sumatra (Miquel 1862). It was mentioned by Miquel (1856) and Koorders (1912), but both authors merely repeated Dunal’s original description, without referring to any specimens. In Koorders’s (1918) botanical report on the flora of northeastern Sulawesi he lists several widespread and common species (e.g., *Solanum lycopersicum* L., *Solanum melongena* L., *Solanum torvum* Sw., *Solanum tuberosum* L.) as well as two shrubby *Solanum* species for which he did not provide names (“Solanum spec. A” and “Solanum spec. B”). Two previously undetermined Koorders collections of *Solanum poka* from northeast Sulawesi (Minahasa Regency) in April 1895 (*Koorders 18035B* and *Koorders 18037B*, both L) correspond to *Solanum poka*. It is possible that these two collections correspond to one (or both) of Koorders’ (1918) unnamed species, but since he provided no descriptions or specific localities this is difficult, if not impossible, to ascertain.

Based on morphology, *Solanum poka* belongs to the *Torva* clade (sensu Stern et al. 2011), with its straight prickles, many flowered inflorescences and corollas with abundant interpetalar tissue (see Fig. 1b). This hypothesis is corroborated by the molecular data (Aubriot et al. 2016). *Solanum poka* is sister to a clade composed of four native Old World species (*Solanum dammerianum* Lauterb. & K.Schum, *Solanum peikuoense* S.S.Ying, *Solanum pseudosaponaceum* Blume, *Solanum torvoideum* Merr. & L.M.Perry) with which it forms a strongly supported group, the ‘Old World torvoids’ *sensu* Aubriot et al. (2016). Morphologically, *Solanum poka* most closely resembles *Solanum pseudosaponaceum*, a widespread species from Taiwan and southern China to Indonesia, but differs in having denser indumentum on the adaxial leaf surface, more numerous straight prickles on the upper stems, fewer, larger flowers with elongate strongly keeled calyx lobes, and much larger fruits. Flowers of *Solanum pseudosaponaceum* are lilac or purplish-white while those of *Solanum poka* are always described on labels as white.

In the protologue Dunal referred to an illustration made by Node-Véran, ‘*Dun. Suppl. 7. Sol. Mss. tab. 55*’, but cited no herbarium material. Similarly to the situation of *Solanum graciliflorum*, we were unable to find any herbarium material matching the illustration in either P or MPU, although Dunal later (Dunal 1816, 1852) cited Leschenault as the collector of the material he had seen. We designate the unpublished illustration of Node-Véran as the lectotype because it is the only extant original material we have identified to date. We designate here an epitype specimen from Java, the cited type locality, (*Horsfield s.n.*, BM000886306) that best matches Node-Véran’s illustration, particularly with respect to the diagnostic characters for *Solanum poka*; leaf shape, prickle shape and calyx lobe morphology.

We have only seen three specimens of *Solanum poka* from Java, the cited type locality, all collected by Thomas Horsfield, an American physician who collected on Java contemporaneously with Leschenault in the early part of the 19^th^ century (McNair 1942, Van Steenis-Kruseman and Van Steenis 1950). *Solanum poka* is, however, rather broadly distributed across the Malay Archiplago, with the distribution centred on Sulawesi and the surroundings islands (Malaku Islands, Talaud Islands) (Fig. 2). Thorough examination of the extensive holdings in Indonesia (particularly those of the Bogor Botanical Garden Herbarium, BO) and, given the historically extensive natural habitat loss recorded for Java (Margono et al. 2014), additional collecting are both needed to better understand the distribution of *Solanum poka*.

Dunal (1852) cited the herbarium name ‘*Solanum quercifolium* Banks’ taken from a specimen in BM collected by Joseph Banks in Java as part of his treatment of *Solanum poka*. Examination of this sheet (BM000886238) shows it belongs to *Solanum pseudosaponaceum*.

## Supplementary Material

XML Treatment for Solanum graciliflorum

XML Treatment for Solanum poka

## Supplementary materials

Supplementary material 1 List of collections used in the study with full locality and descriptive notes Data type: List of collections in xls format Explanation note: Details of the herbarium material examined for this manuscript (including full locality and descriptive notes). This dataset is made available under the Open Database License (http://opendatacommons.org/licenses/odbl/1.0/). The Open Database License (ODbL) is a license agreement intended to allow users to freely share, modify, and use this Dataset while maintaining this same freedom for others, provided that the original source and author(s) are credited.Xavier Aubriot, Caroline Loup, Sandra Knapp
